# Evidence Based Review of Fitness-to-Drive and Return-to-Driving Following Traumatic Brain Injury

**DOI:** 10.3390/geriatrics1030017

**Published:** 2016-07-07

**Authors:** Lisa Palubiski, Alexander M. Crizzle

**Affiliations:** 1 School of Public Health and Health Systems, University of Waterloo, Waterloo, ON N2L 3G1, Canada; lmpalubiski@uwaterloo.ca; 2 School of Public Health, University of Saskatchewan, Saskatoon, SK S7N 2Z4, Canada

**Keywords:** traumatic brain injury, driving performance, simulator, clinical tests

## Abstract

The purpose of this study was to conduct an evidence-based review to determine predictors of fitness to drive and return to driving in persons with traumatic brain injury (TBI). Relevant databases (MEDLINE/PubMed, CINAHL, Cochrane Library, and SCOPUS) were searched for primary articles published before June 2016 using MeSH search terms. Using the American Academy of Neurology’s classification criteria, 24 articles were included after reviewing 1998 articles. Studies were rated by class (I–IV), with I being the highest level of evidence. Articles were classified according to TBI severity, as well as types of assessments (on-road, simulator and surveys). There were no Class I studies. Based on Class II studies, only Post-traumatic amnesia (PTA) duration was found to be probably predictive of on-road driving performance. There is limited evidence concerning predictors of return to driving. The findings suggest further evidence is needed to identify predictors of on-road driving performance in persons with TBI. Class I studies reporting Level A recommendations for definitive predictors of driving performance in drivers with TBI are needed by policy makers and clinicians to develop evidence-based guidelines.

## 1. Introduction

Traumatic brain injury (TBI) is caused when the brain is subjected to external mechanical force resulting in altered neurological function (e.g., loss of consciousness, confusion, disorientation). The severity of the injury may range from minor, with few or no lasting consequences, to major, resulting in profound disability or death. The annual incidence of TBI is conservatively estimated at 600/100,000 in North America [1]. Epidemiological studies show that men, as well as the youngest (15–19 years of age) and oldest cohorts (aged 65 and older) are more likely to sustain a TBI [2]. 

While the issue of TBIs are largely related to sports or returning veterans, the side effects have implications for instrumental activities of daily living such as driving. It is well known that TBI can negatively affect cognitive and motor performance [3,4] and lead to the development of mental health issues [5], all of which can impair driving related abilities. Even in mild TBI cases, impaired cognitive and motor function are often detected by standardized clinical tests up to 3 months post injury [6].

Studies have found that patients with TBI, compared to controls, perform more poorly on hazard perceptions tests [7], have slower information processing speed and reaction time [8]. Recently, one study found that 16.7% of 3993 adults aged 18–97 had suffered a TBI, and compared to licensed older adults without TBI, those with TBI had significantly greater odds of being an aggressive driver in the past 12 months, as well as being in a motor vehicle collision [9]. TBI patients may also have lower self-efficacy, related to fewer kilometres driven, increased use of compensatory driving strategies, driver mistakes and increased inattention [10]. 

Other studies have focused on predictors of driving resumption, often defined as driving status or return to driving. For example, one study found that on-road driver rehabilitation followed by on-road re-assessment was associated with a higher probability of return to driving after TBI [11]. Post-traumatic amnesia (PTA) duration, along with the presence of visual and physical impairment classified 88% of the pass group and 71% of those needing rehabilitation [11]. Due to the impairments associated with TBI, patients are advised against resuming driving for at least 3 months post-injury to allow for appropriate recovery of motor and cognitive abilities [12]. 

Other studies have examined predictors of driving performance in persons with acquired brain injury and found that measures of injury severity [13] and neuropsychological tests [14,15,16] may be useful predictors of on-road driving performance. However, these studies are comprised of mixed samples (e.g., TBI and stroke) that did not control their findings for diagnosis. Consequently, due to differences in etiology (e.g., TBI and stroke) and associated impairments, it is challenging to identify whether predictors of on-road driving are indeed relevant solely to a TBI population. 

A prior evidence based review on TBI and driving that included 13 studies was conducted in 2009. The findings did not support the use of any particular clinical test to predict fitness to drive post-injury in part due to the limited number of on-road studies [17]. Additionally, a recent systematic literature review of the methods and assessment used to determine fitness to drive found that several standardized assessments may be used in TBI patients; however, these tests required further psychometric testing [18]. Additionally, only seven studies were included in this review. In the last decade, there have been an increasing number of studies on TBI and driving. This provides an opportunity to re-assess and summarize the literature to determine whether there are predictors of fitness or drive or predictors of return to driving. Thus, the purpose of this study was to conduct an evidence-based review to assess predictors of fitness-to-drive and return-to-driving in persons with TBI, to identify gaps and provide recommendations for future research. 

## 2. Methods

### 2.1. Procedure

**Figure 1:**
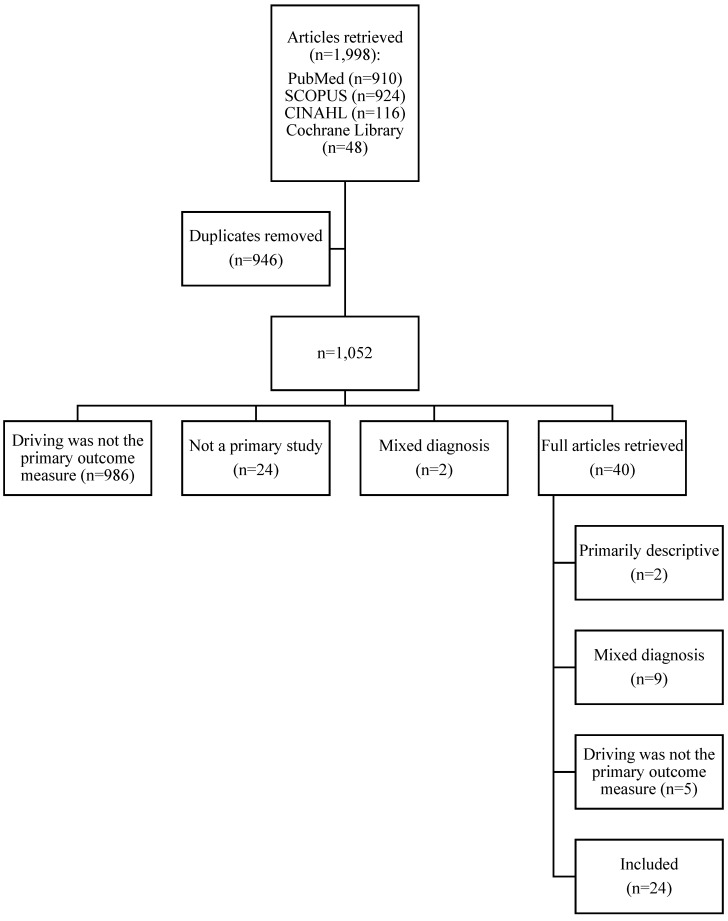
Flow chart of article selection process.

We reviewed primary studies that addressed fitness-to-drive and/or return-to-driving after TBI. To conduct the review, a reference librarian was consulted. Four electronic databases (e.g., MEDLINE/PubMed, CINAHL, Cochrane Library, and SCOPUS) were searched, representing medicine, health science, psychological, and social science. Search terms were as follows: mild, moderate or severe brain injury, concussion (or concussive) and drive (or driving), motor vehicle, or on-road. Also included in the search were the following MeSH headings: brain concussion and automobile driving. In addition, articles were identified via footnote chasing (i.e., finding additional citations in the reference list of selected articles). Articles were excluded for analysis if they (1) were duplicates; (2) were not primary studies; (3) were qualitative or descriptive in nature; (4) included driving, but not as a primary outcome variable; or (5) included mixed diagnosis groups (e.g., TBI and post-traumatic stress disorder). The search included all articles up to June 2016 and yielded 1998 articles (shown in Figure 1). After removing duplicates, 1052 remained; 40 articles were included for full-text review. Twenty-four articles met the inclusion and exclusion criteria. Two independent reviewers extracted the data from each article, with any differences resolved by collaborative discussion. 

### 2.2. Evidence-Based Ratings and Recommendations

The American Academy of Neurology (AAN) classification criteria were used to assign levels of evidence and provide recommendations for studies that examined fitness-to-drive or return-to-driving after TBI [19]. As shown in Table 1, we used the following parameters for rating an article by class (I–IV, with Class I being the highest level of evidence) and recommendation (A-C and U, with A being predictive or not of the outcome, B being probably predictive or not of the outcome, C being possibly predictive or not of the outcome, and U representing inadequate data or conflicting data).

## 3. Results

### 3.1. Description of Primary Studies

Twenty-four studies were included that examined fitness-to-drive [7,8,9,11,20,21,22,23,24,25,26,27,28,29,30], return-to-driving [12,30,31,32,33,34] or both [35,36,37,38] in persons with TBI. The studies included either an on-road driving assessment [8,11,21,22,23,24,29], simulated driving assessment [20,30], off-road screening test [7,25] or self (or other) report measures [9,12,26,27,28,31,32,33,34,35,36,37,38]. The 24 studies that met the inclusion criteria were published before June 2016. Funding status was reported in 14 studies [9,11,12,20,23,24,28,32,33,34,35,36,37,38]. Included studies were conducted in Australia [7,11,12,25,26,29], Canada [9,20,30,34], Europe [8,21,31,36,38] or United States [22,23,24,27,28,32,33,35,37].

### 3.2. Level of Evidence, Conclusions, and Recommendations

A summary of the 24 primary studies are included in the Table S1, including title, year, authors and funding, as well as the study purpose, sample description, independent and dependent variables, study design, main findings, levels of evidence and conclusions. Based on the AAN classification criteria [19] outlined in Table 1, the results, conclusions and recommendations related to the predictors of driving performance in individuals with TBI are presented below. 

#### 3.2.1. On-Road Studies

*Results:* The review yielded five class II studies [8,11,22,23,29] and two class III studies [21,24]. The sample sizes ranged from nine [8] to 207 [11] individuals with TBI. Studies included individuals with mild to severe TBI [11,23,29], moderate to severe TBI [22,24], severe TBI [9] or did not indicate injury severity [21] with a time (mean) post-TBI that ranged from eight months [22] to 12.7 years [23]. The design of the seven studies were prospective [8,22], cross-sectional [21,23,24] or retrospective [11,29] approaches. 

*Conclusions:* One class II study found that poorer performance on a driving simulator was predictive of on-road driving in individuals with moderate to severe TBI [22]. One class II study found that driver distraction (selecting a CD, radio tuning or coin sorting) did not significantly impair on-road driving in persons with mild to severe TBI compared to controls [23]. Two class II studies found that PTA was predictive of pass/fail on the road test [11,20] while one Class II study found that the presence of physical and/or visual impairment, and slower reaction time predicted failing the on-road test [11]. Scores on the Glasgow Coma Scale (GCS) were not predictive of passing/failing a road test [11], as was reaction time [8]. One class III study found that younger age and slower times on the Trail Making Test (TMT) Part B and Useful Field of View (UFOV) Subtest-2 test predicted failing a road test; whereas TMT Part A did not [24]. One class III study found that scores on the perceptual speed test symbol substitution subtest of the Wechsler Adult Intelligence Scale (WAIS), and time estimation task was significantly correlated with the outcome (pass/fail) of the on-road driving task [21]. 

*Recommendations:* Level B: PTA duration is probably predictive of on-road driving performance. Level C: Visual/physical impairment and performance on a driving simulator are possibly predictive of on-road driving performance. Conversely, GCS scores are possibly not predictive of on-road driving performance. Level U: There is mixed evidence of the effect of reaction time on on-road driving performance. The evidence concerning age, perceptual speed, reaction time, symbol substitution, time estimation, TMT A & B and UFOV 2 cannot be interpreted due to inadequate data based on one class III study.

#### 3.2.2. Simulator Studies

*Results:* The review yielded one class III study [20] and one class IV study [30]. The sample sizes ranged from one [30] to 44 [20]. The class III study included individuals with moderate to severe TBI with a time (mean) post-TBI of 53 months [20]. The study design was cross-sectional [20]. The class IV study included one individual with severe TBI, followed over four months of training [30]. 

*Conclusions:* One class III study [20] found no differences in the simulated driving performance between persons with moderate to severe TBI and controls. One class IV study [30] assessed a single case on the driving simulator. Consequently, no conclusions can be made. 

*Recommendations*: Level U: There is no data (or evidence) due to the limited number of studies examining simulated driving performance. 

#### 3.2.3. Off-Road Screening Tests

*Results:* The review yielded two class III studies [7,25]. The sample sizes ranged from 55 [25] to 85 [7]. The first off-road study included individuals with mild TBI and had a time (mean) post-TBI of 10.2 h [7]. The second off-road study included individuals with mild to severe TBI and had a time (mean) post-TBI of 266.4 days [7]. 

*Conclusions:* Two class III studies examined whether GCS scores were associated with the hazard perception test (HPT), an indicator of driving performance [7,25]. Both studies found that scores on the HPT were significantly worse in the TBI group than controls, however, GCS scores were not related with performance on the HPT (response time). 

*Recommendations:* Level C: GCS scores from two consistent Class III studies are possibly not predictive of off-road screening tests. 

#### 3.2.4. Surveys/Self-Report

*Results:* The review yielded 13 primary studies that included survey studies including outcomes related to driving status [12,31,32,33,34,35,36,38], driving behaviours [9,26,27,28,35,36,37] or driving records [27,28,35,38]: all 13 were classified as Class III studies [9,12,26,27,28,32,33,34,35,36,37,38]. The sample sizes ranged from 17 [37] to 4628 [32]. The studies included individuals with mild TBI [12,27], severe TBI [31], mild to severe TBI [26,35] or moderate to severe TBI [32,33,36]. Four studies did not describe injury severity [9,28,34,38]. Time (mean) post-TBI ranged from 30.7 h [12] to 7.1 years [27]. The design of the 13 studies were prospective [12,37], cross-sectional [9,32], retrospective [26,27,28,31,32,33,34,35,36,38], or mixed (prospective and retrospective components) [35] approaches. 

#### 3.2.5. Return to Driving (Driving Status)

*Conclusions:* One Class III study found that lower GCS scores were related to return to driving (driving status) at one, two, and five years post-TBI in persons with moderate to severe TBI [32] However, another Class III study found that GCS scores were not related to driving status, where GCS scores did not differ significantly between the driving and non-driving TBI groups [35]. Two class III studies found that TMT A and TMT B test scores were related to return to driving [34,38] whereas the WAIS Digit span forward or backward was not in one Class III study [34]. One Class III study found that higher scores (indicating better performance) on the Matrix Reasoning Test on the WAIS was related to driving status (more likely to drive) [35]. Two Class III [32,36] studies found that higher scores on the Functional Independence Measure (FIM)—Functional Assessment Measure (FAM; indicating less severity) were related to an increased likelihood of driving, with higher scores on the physical subscale (indicating a physical and motor functionality above 80%) predicting return to driving in another Class III study [31]. One class III study [12] found that time to complete the occupational therapy-drive home maze test was predictive of return-to-driving 2 weeks after sustaining a TBI. In a class III study [33], directives against driving from significant others (e.g., caregiver perceptions) were related to non-driving. 

*Recommendations:* Level C: FIM, TMT A and B scores are possibly predictive of driving status based on two Class III studies; Level U: Evidence concerning GCS score, WAIS, Drive Home Maze test and caregiver perceptions cannot be interpreted due to inconsistent or inadequate data with each based on one class III study. 

#### 3.2.6. Driving Behaviours

*Conclusions:* One class III study found that GCS scores (indicating less severe injuries) were associated with greater driving exposure (e.g., drive more frequently and over greater distances) and lower driving avoidance (less likely to drive with passengers, in busy traffic, at night and on the freeway) [26]. Shorter PTA durations (indicating less severe TBI) were associated with greater driving exposure in two Class III studies [26,37], as was a higher score (indicating less severe injury) on the Digit Span Subtest on the WAIS in one Class III study [37]. Conversely, the FIM Motor Subscale was predictive of restricted driving exposure [37]. 

Evidence concerning PTA with driving avoidance was inconsistent. One study found a negative correlation [26], whereas the other study found that PTA durations did not show a significant effect on driving avoidance [37]. The FIM was not associated with driving avoidance in one Class III study [37].

*Recommendations:* Level C: PTA duration is possibly predictive of driving exposure; Level U: Driving exposure: Evidence concerning GCS, WAIS and FIM scores cannot be interpreted due to inadequate data. Driving avoidance: Evidence concerning PTA duration and FIM scores cannot be interpreted due to conflicting or inadequate data based. 

#### 3.2.7. Driving Records 

*Conclusions:* One Class III study found that the processing speed index on the WAIS, as well as scores on the TMT A and B tests were not predictive of self-reported crashes or citations in individuals with mild TBI [27]. One class III study [38] found that the number of years post-TBI, personality index scores from the Diagnostic and Statistical Manual of Mental Disorders 4th Edition (higher scores indicate more risky attitudes and behaviours) and driving-style index scores (higher scores indicate more risky driving practices) were positively associated with accidents and violations. Two class III studies [9,28] compared the number of accidents and violations between those with TBI and controls. One study found that the TBI group had significantly more on-road collisions after TBI whereas the other study did not [28]. 

*Recommendations:* Level U: Evidence concerning WAIS, Trail Making A & B scores, number of years post TBI, personality and driving index scores and self-reports of accidents are inconclusive due to inadequate or conflicting data. 

## 4. Discussion 

This review found 24 primary studies examining TBI and driving highlighting the limited number of studies in this area. Overall, there was substantial variation between studies in sample characteristics (e.g., TBI severity), sample size, the clinical tests performed, outcome measures, as well as the length of follow-up periods, making it difficult to draw definitive conclusions and concise recommendations. Similar to a prior review [18], we found no standard clinical battery to predict driving performance in persons with TBI although a few individual tests may warrant further consideration in future studies. 

From the on-road studies, injury severity (PTA duration) was the only probably predictor. While PTA duration and GCS scores are both measures of injury severity; they produce different classifications of TBI severity. For example, in one study that used both measures [11], only 2% of participants were classified in the same category (mild, moderate and severe), which may explain why GCS scores were not related to any outcomes in the present study (e.g., on-road and simulator performance, driving behaviours, crash records). 

While one study found that driving performance on a simulator may be indicator of on-road driving performance [11], another study found no differences in simulated driving performance between those with moderate/severe TBI and controls [20]. Consequently, there is no evidence of impaired driving in those with TBI on a simulator. While the on-road test is often regarded as the ‘gold standard’ to examine fitness to drive; pass/fail outcomes are often needed to determine whether driving performance is actually impaired. A driving simulator, meanwhile, especially one with high fidelity, may allow researchers to evaluate fitness-to-drive in persons with TBI. There have been a few studies that have used simulators as a proxy to on-road driver testing with good relative and absolute validity [22,39,40]. Driving simulators may also offer opportunities to test a wider range of capabilities by testing persons with TBI on more challenging tasks that are not performed on the road (e.g., driving in bad weather, driving at night, driving in high traffic) [22]. In some jurisdictions, a simulator can be part of a tiered approach to driving assessment where persons who fail on the simulator are then referred for an on-road test. However, there were few simulator studies and consequently, no recommendations could be made concerning the predictive nature of any clinical test or simulator assessment in persons with TBI. 

Other well-known clinical tests such as the Trails B and UFOV, which are often predictive of driving performance in older drivers and other clinical populations [41,42], were both predictive of on-road pass/fail outcomes whereas the Trails A was not. As deficits related to TBI often impair executive function and information processing speed, further studies are needed to determine whether the Trails B test or UFOV, as well as other clinical tests, can either individually or be combined into a clinical battery for screening. 

No predictors related to off-road screening tests were found besides the GSC being possibly not predictive in relation to the hazard perception test [7,25]. FIM, Trails A and B scores are possibly predictive of return to driving and PTA duration is possibly predictive of driving exposure. Recommendations concerning other tests (e.g., WAIS, GCS, Trails A & B scores, Drive Home Maze Test) on return to driving, driving exposure (GCS, WAIS and FIM), driving avoidance (PTA, FIM) and driving records (e.g., WAIS, Trails A & B, number of years post TBI, personality and driving index scores and self-reports of accidents) were all inconclusive. While return to driving was consistently assessed using surveys, it should be noted that there are limitations to using self-report measures, which include social desirability and recall bias [43,44,45]. Additionally, crash records may not be a true reflection of crash risk as minor crashes may not be reported to insurance companies or reported by police [34]. Crashes in general also happen infrequently. Future studies should attempt to follow drivers with TBI over time to determine predictors of return to driving (via an on-road test), as well as rehabilitation protocols employed by occupational therapists (or other rehabilitation specialists) that enhance the likelihood of return-to-driving following a TBI. To date, there is little information on intervention protocols and effects on return-to-driving in persons with TBI.

Limitations in the field include the substantial variability in outcome measures and assessment procedures (including length of monitoring). When patients are recruited and assessed, and whether there is a follow-up period, are important considerations in future studies. Two studies found driving performance was impaired 24 h of mild TBI onset [7,12] while another study found no impairment years after TBI diagnosis [27]. This suggests mild TBI may not result in residual impairment but may result in impairment soon after injury. Adequate screening is important given that 60%–80% of all TBI cases are considered mild and many deficits often go unrecognized and untested, yet pose a significant danger to road safety. Developing screening measures to identify those with mild TBI is critical to ensuring road safety (and the safety of other road users) despite the limited number of studies available.

These findings support the notion that Class I studies with Level A recommendations are needed to develop clear and concise evidence based guidelines for assessing fitness to drive in TBI populations. This study could only make limited recommendations due to small and heterogeneous sample sizes, which may impede the ability to detect small and moderate effects, both within and between groups. Future studies should attempt to recruit larger and more homogenous samples and examine difference related to mild, moderate and severe TBI on driving performance. 

## Figures and Tables

**Table 1:** Ratings and Recommendations by American Academy of Neurology (AAN) criteria.

	Class I	Class II	Class III	Class IV
Rating article by class	Evidence provided by a prospective study in a broad spectrum of persons with the suspected condition, using a criterion standard for the case definition. Test should be applied in a blinded evaluation. All people undergoing the test have the presence or absence of the condition.	Evidence provided by a prospective study of a narrow spectrum of persons (n, 100) with the suspected condition, or a retrospective study of a broad spectrum of persons with an established condition by criterion standard, compared to a broad spectrum of controls.	Evidence provided by a retrospective study where either persons with the established condition or controls are of a narrow spectrum (n, 100). The reference standard, if not objective, is applied by someone other than the person performing the test.	Any design where the test is not applied in an independent evaluation or evidence provided by the expert opinion alone or in descriptive case series (without controls).
	**Level A**	**Level B**	**Level C**	**Level U**
Rating by recommendation	Recommendation: Established as effective/useful/or predictive or not. “Should be done, or should not be done.”	Recommendation: Probably effective/useful/or predictive, or not. “Should be considered, or should not be considered.”	Recommendation: Possibly effective/useful/or predictive, or not. “May be considered, or may not be considered.”	No recommendation.
Condition for rating by recommendation	Requires 2 consistent Class I studies, or 1 Class I study where the magnitude of the effect is large, and all criteria have been met.	Requires at least 1 Class I study, or 2 consistent Class II studies.	Requires at least 1 Class II study, or 2 consistent Class III studies.	Data inadequate or conflicting. Given the current knowledge or test, the treatment is unproven.

## Supplementary Materials

The following are available online at http://www.mdpi.com/2308-3417/1/3/17/s1, Table S1: Summary of Included Primary Studies.
