# Supplementary material for: Evidence Based Review of Fitness-to-Drive and Return-to-Driving Following Traumatic Brain Injury
Source: Geriatrics (Basel). 2016 Jul 7;1(3):17. doi: 10.3390/geriatrics1030017 (PMC6371138; doi:10.3390/geriatrics1030017)
Supplement: Supplementary file 1 [file geriatrics-01-00017-s001.pdf]

Supplementary Materials: Evidence Based Review of Fitness-to-Drive and Return-to-Driving Following Traumatic Brain Injury

Lisa Palubiski and Alexander M. Crizzle

Table S1. Summary of included primary studies.

	Purpose	Sample	Independent Variable	Outcome Measure	Study Design	Main Findings	Class and Recommendation
				**On-Road Studies**			
Neuropsychological driving fitness tests for brain-damaged subjects Korteling and Kaptein (1996) [1] The Netherlands Funding not described	To examine the use of neuro-psychol ogical tests as predictors of on-road driving performance in brain-injured participants	N = 38; Patients with TBI; 33 men and 5 women; Age (mean) = 29.8 years (SD = 10.9); Time post-injury = at least 1 year; PTA = 33 days (SD = 51 days)	Perceptual Speed test; WAIS Symbol Digit Substitution subtest, Reaction dual task (divided-attention task); Time estimation; driving performance errors	Standardized on-road assessment	Quasi-experimental; Cross-sectional study design	Perceptual speed ($p < 0.05$) and time estimation ($p < 0.05$) were predictors of on-road driving performance (significant, but low correlation); Perceptual Speed, Symbol Substitution, and Time Estimation tests (combined) predicted 35.3% of variance of the on-road driving performance (pass/fail outcome of on-road driving evaluation)	Class II: Quasi-experimental; Cross-sectional; N < 100 Conclusion: No single test can be used to replace on-road driving assessments; perceptual speed and time estimation may be used as part of a standard battery to assess for fitness to drive
Predictive validity of driving-simulator assessments following traumatic brain injury: A preliminary study Lew et al. (2005) [2] USA Funding not described	To examine driving simulation and road test evaluations as predictors of long-term driving performance in individuals with moderate to severe TBI	N = 27; Patients (n = 11) with moderate to severe TBI; 82% male; Age (mean) = 29 years (SD = 12); Time (mean) post-injury = 8 months (SD = 2, range 2 to 25 months); Patients recruited from referrals to driver evaluation program; Healthy subjects (n = 16); 75% male; Age	Baseline: Simulator (STI® version 8.16) evaluation: 1. SPI (automated measures): Speed control measures (speed, speed variability, acceleration variability, speed jerk, and red-light violations) and direction-control measures (lane position error on straight roads, lane position variability on curved roads, steering jerk, collisions, and deviations off-road) 2. DPI (observational	10 months: 1. On-road assessment: DPI (observational data) 2. Report of driving infractions (self-report)	Quasi-experimental case-control study design; Prospective	55% of patients with TBI failed the simulator trial (two standard deviations below the mean of the healthy reference group) at baseline, while healthy subjects (all) passed; At baseline, patients with TBI performed poorer on SPI ($p = 0.001$), compared to healthy control drivers and were impaired on speed ($p = 0.001$), speed variability ($p < 0.001$), acceleration variability ($p = 0.004$), lane position variability (straight	Class II: Quasi-experimental case-control study design; Prospective; N < 100; With blinding (Observers at ten months were blind to measures at baseline); On-road assessment Conclusion: Simulator-based assessment provides a valid measure of long-term driving performance, and may be more sensitive than

		(mean) = 36 years (SD = 11)	data): Handling of controls (steering wheel control, and throttle-brake coordination), regulation of trajectory (speed, lane tracking and brake reaction time), basic maneuvers (lane changes, execution of turns, merging into traffic, obedience to traffic signs and signals, and following distance) and higher-order skills (safety judgements when passing and yielding right of way, speed and correctness of decisions, and emotional stability and self-control) On-road assessment 3. DPI (observational data)		road, $p = 0.02$), steering jerk ($p = 0.02$), collisions ($p = 0.003$), deviations off road ($p = 0.04$), and divided attention task (hits versus misses, $p < 0.001$); At baseline, patients with TBI also performed poorer on the observational measures of simulator performance (DPI), and were impaired on all four subscales (handling of controls, regulation of trajectory, basic maneuvers, and higher order skills, all $p < 0.01$); Automated SPI measures, at baseline, significantly predicted observational DPI at ten months, and predicted handling of controls ($p = 0.002$), regulation of trajectory ($p = 0.02$), and higher-order skills ($p = 0.73$); Simulator observations (SPI), at baseline, also significantly predicted observational DPI at ten months, and predicted higher-order skills ($p = 0.04$); Simulator trial (SPI and DPI) showed overall predictive validity of 82% (100% sensitivity and 71% specificity); Road test DPI score at baseline was not associated with DPI score with driving performance at ten months	an on-road assessment in patients with moderate to severe TBI

Predictors of the on-road driving assessment after traumatic brain injury: Comparing cognitive tests, injury factors, and demographics<br>McKay et al. (2015) [3]<br>Australia<br>Funding not described	To examine the ability of cognitive tests to predict on-road driving performance	N = 97; Patients with mild to severe TBI; 86% male; Age (mean) = 40.6 (SD = 14.8); GCS (mean) = 8.7 (range = 3 to 15); PTA duration (mean) = 23.5 days (SD = 21.9); Recruited from database of patients with TBI who completed a driving assessment	Demographics (sex, age, and years licensed); PTA duration; Time post-injury; Wechsler Test of Adult Reading or National Adult Reading Test-Revised (RAVLT); RCFT; WAIS-III (Block Design subtest, Similarities subtest, Digit Span subtest, Digit Symbol-Coding subtest); TMT B;	On-road driving assessment: Pass/Fail outcome	Retrospective case series	21% of patients failed the on-road test; Compared to the pass group, the fail group performed significantly poorer on the RCFT, Digit Span, Block Design, Similarities, and TMT B ($p < 0.05$); Compared to the pass group, the fail group had significantly longer PTA duration and time post-injury; Although significant, cognitive tests were poor predictors of the driving assessment (poorly correlated); PTA duration was found to be a strong predictor of a pass/fail on the road test(r = 0.29, p = 0.04)	Class II; Retrospective study; N < 100; On-road assessment Conclusion: Cognitive tests were found to be poor predictors of the on-road driving assessment
The effects of driver distraction for individuals with traumatic brain injuries<br>Neyens et al. (2015) [4]<br>USA<br>Funded by the National Institute of Child Health and Human Development	To examine the effects of secondary tasks on on-road driving in TBI and healthy control drivers	N = 39; TBI drivers (n = 16); 5 women and 11 men; Age (mean) = 37.5 years (SD = 11.4); 1 with mTBI, 3 with moderate TBI, and 9 with severe TBI; Time (mean) post-injury = 12.7 years (SD = 9.3); Recruited from rehabilitation facilities and from database of individuals who had participated in other research studies; Healthy control drivers (n = 19); Age (mean) = 38.7 years (SD = 12.4)	Secondary tasks performed during on-road assessment: 1. Selecting a CD 2. Radio tuning 3. Coin sorting	1. Driving performance (on-road assessment): Mean speed, standard deviation of mean speed, and maximum lateral acceleration 2. Eye gaze behaviour: Percentage of time looking at the task, the number of glances, and the duration of the longest glance	Quasi-experimental case-control study design; Cross-sectional	35.2% of participants reported that they had attended driving training after TBI; 41.2% of participants reported that they had changed driving habits after TBI; No significant difference between groups in time to complete the task (selecting the CD, radio turning, and coin sorting) and in measures of driving performance; Drivers with TBI spent significantly more time looking at the secondary tasks and looked more frequently at the tasks ($p < 0.05$)	Class II: Quasi-experimental case-control study design; N < 100; Cross-sectional Driving evaluator was blinded; On-road assessment Conclusion: Drivers with TBI were significantly more likely to glance and to glance for longer periods of time, compared to healthy control drivers, when completing a secondary task, which may compete with resources needed for driving and impair driving; however, no differences in driving performance were observed

UFOV performance and driving ability following traumatic brain injury<br>Novack et al. (2006) [5]<br>USA<br>Funded by the National Institute of Health Grant	To examine the relationship between the UFOV and driving performance after moderate to severe TBI	N = 60; 22 women and 38 men; Age (mean) = 30 years (range 16 to 68); Time (mean) post-injury = 17.5 months (range 2 months to 19 years); 18% with moderate TBI and 72% with severe TBI; (based on GCS score of 29 of the subjects); Referred by physician	UFOV (Visual Attention Analyser, Model 2000): Three sub-tests of increasing difficulty (Processing speed, divided attention, and selective attention); TMT A and B	1. GRS (on-road assessment of driving performance); Rated 0–3, with 0 representing 'should not be driving under any conditions' and 3 representing 'able to drive in any conditions'<br>2. DAS (checklist of 25 driving behaviours; secondary measure of driving performance; completed during evaluation); Checklist includes, maintaining speed, signalling lane changes, and braking smoothly	Experimental study design; No control	Higher DAS scores associated with more favourable driving evaluation outcomes ($p < 0.01$); DAS scores associated with the second ($p < 0.01$) and third ($p < 0.05$) UFOV subtests; Age (where younger participants performed worse, $p < .01$) performance on TMT B ($p < .05$) and higher score on UFOV subtest two found to be significant predictors of a failure (not cleared to drive under any circumstances) rating by the driving instructor; Age (where younger participants performed worse, $p < 0.01$) and higher score UFOV subtest two ($p < 0.05$) predicted poor observer-rated DAS scores	Class III: Experimental study design; Cross-sectional; N < 100; Driving evaluator blind to UFOV test score; No control; On-road assessment<br>Conclusion: UFOV performance and TMT B (but not TMT A) performance found to be predictive of on-road driving performance

Predictors of on-road driver performance following traumatic brain injury<br>Ross et al. (2015) [6]<br>Australia<br>Funded by the Royal Automobile Club of Victoria Sir Edmund Herring Memorial Scholarship	To examine the predictors of passing an on-road driving assessment after mild to severe TBI	N = 207; Patients with mild to severe TBI; 156 men (68.4%); PTA (mean) duration = 23.34 days (SD = 25.51); 2% mild, 31.3% moderate, and 35.3% severe; GCS (mean) = 9.6 (SD = 4.3); 42% mild, 14.9% moderate, and 43.1% severe); Recruited from rehabilitation hospital	GCS score; PTA duration; Physical and/or visual impairment; Reaction time	On-road driver assessment: Pass/Fail outcome	Cohort study design; Retrospective	66% (n = 137) passed driver assessment and 34% (n = 70) failed (in rehabilitation (or failed) group, 94% of group returned to driving after driver rehabilitation); Significant predictors of the pass group include being male, having shorter PTA duration, no physical and/or visual impairment, and faster reaction time ($p <$ 0.001); Variables correctly classified 87.6% of the pass group and 71.2% of the rehabilitation group	Class II: Retrospective study; N > 100; On-road assessment Conclusion: PTA duration proved to be better predictor of driver assessment outcome than GCS score; PTA (with the presence of impairment and slower reaction time) could be used as criteria to refer patients to driver assessment
Task and driving performance of patients with a severe concussion of the brain<br>Stokx & Gaillard (1986) [7]<br>Holland<br>Funding not described	To examine reaction time as a predictor of on-road driving performance in persons with severe concussions	N = 9; Patients with severe concussion; Age (mean) = 25 years (SD = 3); Patients tested 2 years after sustaining concussion<br>Age and gender matched control group (N = 9); Demographics not specified)	Reaction time	On-road driving performance (braking, speed regulation, secondary distraction)	Quasi-experimental case-control study design; Prospective	Patients made more overall errors than controls but differences not significant. Patients had significantly slower reaction time than controls when braking, shifting gears and performing secondary tasks ($p < 0.05$) but passed all tests.	Class II: Prospective study; N > 100; On-road assessment with control group. Conclusion: No conclusive evidence that reaction time impairs driving performance.

Table S1. *Cont.*

	Purpose	Sample	Independent Variable	Outcome Measure	Study Design	Main Findings	Class and Recommendation
				**Simulated Driving Studies**			
Attention following traumatic brain injury: Neuro-psychological and driving simulator data, and association with sleep, sleepiness, and fatigue Beaulieu-Bonneau et al. (2015) [8] Canada Funded by the Canadian Institutes of Health Research	To assess driving simulation performance in individuals with moderate to severe TBI	N = 44; Individuals with moderate to severe TBI (n = 22); GCS (mean) score = 7.2 (SD = 3.6); Time (mean) post-injury = 53 months (SD = 37.1); Recruited through medical records, referrals, and solicitation of members of an association of TBI survivor; Matched (gender, age, and education) controls (n = 22)	TBI	D-KEFS TMT (variation of the original TMT; consisting of five subtests; completion time and score): Visual scanning (TMT-1), number sequencing (TMT-2, similar to TMT B), letter sequencing (TMT-3), number-letter switching (TMT-4, similar to TMT A), and motor speed (TMT-5); Driving simulation performance (STISIM Drive™): Number of minor infractions, variability of lateral position (SD of the vehicle lateral lane position), mean speed, speed variability (SD); mean hit reaction time	Quasi-experimental case-control study design; Cross-sectional	Significant difference between groups for TMT 1, 2, 4, and 5 ($p = 0.03$), with individuals with TBI displaying longer completion times; Significant difference in lateral position, with individuals with TBI displaying greater variability ($p < 0.01$)	Class III: Quasi-experimental case-control design; Cross-sectional; N < 100; Driving simulator Conclusion: In the TBI group, poorer performance on D-KEFS TMT (longer completion times) was observed, suggesting tapping speed of information processing is affected years after moderate to severe TBI and may impact driving performance

In-simulator training of driving abilities in a person with a traumatic brain injury<br>Gamache et al. (2011) [9].<br>Canada<br>Funding not described	To examine in-simulator training program after severe TBI	N = 1; 23 year old woman; Approx. 5 years after TBI; PTA duration = 12 days; Training lasted 4 months (25 visits; drove in up to three different scenarios each visit; drove roughly 54 km per visit, taking roughly 58 min to complete)	Static visual acuity; MVPT; Bells test; TMT A and B (time); Foot choice reaction time; Active range of motion; Manual muscular strength	Driving simulation performance STISIM Drive 2.0: Lateral position of the car; Deceleration profiles at intersections; RT to an auditory signal	Case study; 1 year follow-up	Data collected on 12 of the 25 visits (divided into early and late training) and at the retention session (1 year follow-up); Significant improvement in lateral position seen at late training ($p < 0.001$); however, improvement was not retained; No significant difference in standard deviation of lateral position seen in late training; Reduced cognitive load (RT to an auditory signal) seen at late training ($p < 0.001$), and maintained at 1 year follow up; Less "jerky" speed profile found when decelerating ($p < 0.001$), and performance maintained at 1 year follow up	Class IV: Case study; Prospective; Driving simulator Conclusion: Simulator-based education program proved to be useful in driving rehabilitation after severe TBI

Table S1. *Cont.*

	Purpose	Sample	Independent Variable	Outcome Measure	Study Design	Main Findings	Class and Recommendation
**Off-Road Screening Studies**							
Driving after concussion: The acute effect of mild traumatic brain injury on drivers' hazard perception Preece et al. (2010) [10]. Australia Funding not described	To examine drivers' hazard perception after mTBI	N = 85; Patients with mTBI (n = 42); 6 women and 36 men; Age (mean) of patients with mTBI = 25.4 years (SD = 7.5); GCS (mean) score = 14.8 (SD = 0.5); Time (mean) post-injury = 10.2 h (SD = 5.2); Recruited from emergency department; Minor orthopedic injuries, control group (n = 43); 11 women and 32 men	Spatial RT Task; Pain and Emotionality Scale; DASS-21; Digit Symbol Substitution Test; GOAT; Manchester DBQ; Driving questionnaire; Recovery questionnaire; National Adult Reading Test (2nd ed.); LogMar Visual Acuity Test	University of Queensland HPT	Quasi-experimental case-control study design; Cross-sectional	Significant difference in response to traffic hazard (*p* = 0.03) where patients with mTBI were slower to respond to hazards, compared to control group; GCS scores were not significantly correlated with HPT response times	Class III: Quasi-experimental; Cross-sectional; N < 100; Screening test Conclusion: mTBI was associated with impairment on driving-related tasks (hazard perception) in the first 24 h' post-injury
Assessment of drivers' ability to anticipate traffic hazards after traumatic brain injury Preece et al. (2011) [11]. Australia Funding not described	To examine driver's ability to anticipate traffic hazards after TBI	N = 55; Patients with mild to severe TBI (n = 31); 5 women and 27 men; PTA (mean) duration = 44.0 days (SD = 37.0); GCS (mean) score = 8.0 (SD = 4.7); Time (mean) post-injury = 266.4 days (SD = 440.2); Recruited from Rehabilitation Unit; Age-matched uninjured controls (n = 24); 9 women and 15 men	Spatial RT Task; Digit Symbol Substitution Test; National Adult Reading Test; Manchester DBQ; DASS-21; Alcohol Use Disorders Identification Test; Rivermead Post Concussion Symptoms Questionnaire; Behavioural Identification Test (star cancellation task)	University of Queensland HPT	Quasi-experimental case-control study design; Cross-sectional	Significant difference in response to traffic hazards on HPT, where patients with TBI were slower to respond (*p* < 0.001) compared to control group; Response time related to duration of PTA (*p* < 0.001, but not GCS; Patients with mild TBI significantly faster to respond to traffic hazards than patients with moderate to severe TBI (*p* = 0.04)	Class III: Quasi-experimental; Cross-sectional; N < 100; Screening test Conclusion: Patients with TBI were slower to anticipate traffic hazards on HPT, compared with controls, which may impair driving performance

Table S1. *Cont.*

	Purpose	Sample	Independent Variable	Outcome Measure	Study Design	Main Findings	Class and Recommendation
**Survey Studies**							
Fitness to drive after mild traumatic brain injury: Mapping the time trajectory of recovery in the acute stages post injury Baker et al. (2015) [12]. Australia Funded by La Trobe University Physical Activity and Rehabilitation Group	To examine driving status of patients with mTBI	N = 120; 30 women and 90 men; Recruited from the Trauma and Orthopedic ward at The Alfred Hospital; Patients with mTBI (n = 60); Age (mean) = 39.5 years (SD = 15.4); GCS (mean) score = 14.6 (SD = 0.6); Time (mean) post-injury = 30.7 h (SD = 10.2); Orthopedic injury (n = 60, control group); Age (mean) = 38.23 years (SD=13.32)	MMSE; OT-DHMT; RLRCT; University of Queensland HPT (SSRT, HPT-RT, and HPT-HR) at 24 h post-injury	Driving status (driver or non-driver) at two weeks post-injury	Quasi-experimental case-control study design	No significant difference between mTBI and orthopedic injury group on MMSE, RLRCT, HPT-RT, and HPT-HR at 24 h post-injury; Significant difference between mTBI and orthopedic injury on SSRT ($p = 0.04$) and OT-DHMT ($p = 0.01$) at 24 h post-injury, where patients with mTBI completed the maze slower; 26% of patients with mTBI had returned to driving within two week post-injury; Scores at 24 h post-injury (and injury severity) predicted 31% of variance in time taken to return to driving; 30% of mTBI group reported no issues with return to driving	Class III: Quasi-experimental; Prospective; N > 100; Self-report Conclusion: Patients with mTBI, 24 h after TBI, have impaired driving performance
Predictors of driving outcome post-TBI Coleman et al. (2002) [13]. USA Funded by the National Institute of Disability Research and Rehabilitation, Traumatic Brain Injury Model Systems Project, US Department of Education and Wayne State University	To examine predictors of driving status and fitness to drive after TBI	N = 71 pairs; Participants with moderate to severe TBI (N = 71); 14 women (19.7%) and men (80.3%); GCS (range) score = 3 to 12; Time (mean) post injury = 4.3 years (SD = 2.6); Recruited from Southeastern Michigan TBI System database;	1. Participants with TBI: GCS (Becker conversion; at discharge); DRS (at discharge); WAIS-III letter-number sequencing test; WAIS-III matrix reasoning test; CTT; PCRS 2. Caregivers: PCRS, SPS	Driving status (driver or non-driver); Driving frequency (estimated driving miles per week); Post-injury driving record (official driving record requested from Department of	Single cohort study design; Retrospective and prospective follow-up	46% of patients with TBI returned to driving; Demographic variables (age, gender, etc.) not associated with driving status post-injury; Significant other perceived social support (SPS) was significantly associated with patient driving status (driver or non-driver), where SPS scores (examining perceived social support) were lower for	Class III: Retrospective and prospective; N > 100; Single cohort; Subjective and objective measures Conclusion: Significant other's perceptions of patients' fitness to drive was the strongest predictor of patients' driver status and driving frequency

		Caregivers (N = 71); 52 women (73%) and 19 men (27%)		Motor Vehicles, representing all traffic violations in the patient's' driving history)		non-drivers than for drivers ($p = 0.03$); PCRS rating (by both patient and significant other), specific for driving ability, was greater for drivers compared to non-drivers ($p < 0.01$); Neuropsychological functioning, awareness of deficit, and patients' and significant others' perception of the patient's ability to drive safely correctly classified 80.3% of cases, with 81.6% of non-drivers and 78.8% of drivers correctly classified	
Early neuropsychological tests as correlates of return to driving after traumatic brain injury Cullen et al. (2014) [14]. Canada Funded by the Canada Foundation for Innovation	To assess the predictive ability of neuro-psychological tests administered during inpatient rehabilitation	19 participants with TBI who returned to driving (drivers); Age (mean) = 48.5 (SD = 14.3); GCS (mean) = 7.0 (SD = 3.3); Years (mean) post-injury = 8.3; Participants who returned to driving were case-matched to 19 participants with TBI who had not returned to driving (non-drivers); Matched on age, GCS score, and DRS; Age (mean) = 49.0 years (SD = 14.9); GCS (mean) = 6.5 (SD = 3.9); Years (mean) post-injury = 5.8; Recruited from acquired brain injury database	TMT A and B; WAIS-III Digit span forward and backward	Return to driving (yes/no)	Retrospective; Case-controlled study design	Comparison of neuropsychological test scores between drivers and non-drivers found that drivers score significantly better on TMT A ($p < 0.01$) and TMT B ($p < 0.01$), but not on Digit span forward or backward tests	Class III: Retrospective; n < 100; Case-controlled Conclusion: Processing speed (TMT A) and cognitive flexibility (TMT B) measures may predict return to driving after TBI

Return to driving after head injury<br>Hawley (2001) [15].<br>England<br>Funded by the Warwick Business School	To examine driving status and driving related problems after TBI	N = 563; Age (mean) = 32.5 years (range 12 to 65 years); 77.1% men; GCS score available for 402 (70.8%) patients and PTA duration available for 196 (34.8%) patients: 60 (10.8%) with mild TBI, 120 (21.4%) with moderate TBI, 153 (27%) with severe TBI, and 230 (40.8%) with very severe TBI; Time post injury (range) = 6 months to 2+ years; Recruited from multiple (10) rehabilitation units	FIM + FAM (combined score; at time of interview)	Driving status (driver or non-driver); Driving related problems	Single cohort study design; Cross-sectional	381 (67.7%) drove before TBI; 270 (47.5%) interviewed within 6 months of TBI, 383 (67.5%) within 1 year, 461 (81.9%) within 2 years, and 102 (18.1%) more than 2 years after TBI; 139 (36.5%) of the 381 previous drivers had returned to driving at the time of the interview (current drivers); Of the 381 previous drivers, 41 (10.8%) received driving ban and 20 (5.3%) were advised not to drive; Reported problems among current drivers include anger, aggression, and irritability (67, 48.2%), memory (89, 64%), concentration and attention (39, 28.1%) and vision (39, 28.1%); FIM and FAM combined score significantly higher in current drivers compared to ex-drivers ($p = 0.001$ for attention, orientation, and safety judgement and $p = 0.002$ for emotion); Significant difference in injury severity between current drivers and ex-drivers ($p = 0.001$), such that current drivers sustained less severe TBI	Class III: Cross-sectional, N > 100; Single cohort; Self-report Conclusion: Driving related problems (problems with anger, aggression, and irritability, memory, concentration and vision) does not prevent patients with TBI from return to driving; Current drivers scored significantly higher on the FIM+FAM, and sustained less severe injuries

Traumatic brain injury, driver aggression and motor vehicle collisions in Canadian adults<br>Ilie et al. (2015) [16].<br>Canada<br>Funded by Canadian Institutes of Health Research (STAIR Team Grant, by the Ontario Neurotrauma Foundation, and AUTO21, a member of the Networks of Centres of Excellence program that is administered and funded by the Natural Sciences and Engineering Research Council, the Social Sciences and Humanities Research Council, in partnership with Industry Canada	To examine the relationship between brain injury (lifetime TBI), road aggression, and road collisions	N = 3,993; 41.4% men; Age (mean) = 53.67 years (SD = 16.67, range 19 to 97 years); Patients with head injury (TBI) resulting in loss of consciousness (at least 5 min) or overnight hospitalization (n = 575); Recruited from survey by Center for Addiction and Mental Health	TBI	Driver aggression (past 12 months); Driving collisions (self-report, accidents or collision involving any kind of damage or injury)	Cross sectional study design; Single cohort	91% of participants had valid driver's license; 16.7% of participants with a valid driver's license had a lifetime history of TBI; Relative to group without TBI, group with TBI was more likely to engage in serious driver aggression (AOR = 4.39, $p < 0.01$) and to be involved in a road collision (AOR = 1.74, $p < 0.05$)	Class III: Cross sectional study design, N > 100; Self-report<br>Conclusion: The population-based study found evidence of a relationship between a lifetime history of TBI and higher rates of serious driver aggression and involvement in road collision

Predictors of driving avoidance and exposure following traumatic brain injury Labbe et al. (2014) [17]. USA Funded by the National Institute on Disability and Rehabilitation Research	To examine predictors of driving avoidance and exposure after moderate to severe TBI	N = 184; 55 (29.9) women and 129 (70.1%) men; Age (mean) = 34.9 years (range 16 to 79); GCS (mean) score = 8.7; PTA (mean) duration = 34.9 (SD = 19.9); Participants with moderate to severe TBI; Recruited from TBIMS database	Injury severity (PTA); TMT A (time); TMT B (time); Digit Span subset of WAIS-III; FIM Motor subscale	Self-reported driving exposure (frequency of driving during the past month and the average distance driven per week) and avoidance of driving situations	Single cohort study design; Prospective; 1, 2, and 5 year follow-up	Age and gender related to driving avoidance, where older and female participants avoided a greater number of driving situations (no p-values); Participants with less severe TBI (shorter PTA duration) and greater performance on cognitive measures (TMT A, TMT B, and Digit Span subset of WAIS-III) at discharge had greater driving exposure at follow-up times (drove more frequently and over greater distances); PTA associated with FIM Motor subscale score, where more severe injuries resulting in decreased functional mobility; More severe injury not associated with greater avoidance of driving situations	Class III: Prospective; N > 100; Single cohort; Self-report Conclusion: Older and female participants were more likely to avoid driving situations after TBI, and as expected, those with less severe TBI exhibited greater driving exposure; however, did not avoid a greater number of driving situations
Driving with cognitive deficits: neuro-rehabilitation and legal measures are needed for driving again after severe traumatic brain injury Leon-Carrion et al. (2005) [18]. Spain Funding not described	To examine return to drive after severe TBI, whether post-traumatic cognitive deficits prevent safe return to drive and whether neuro-rehabilitation program improves safe return to drive	N = 17; Patients with severe TBI (GCS (mean) score of 6, SD = 2.54); Age (mean) = 22.9 years (SD = 6.9); Time (mean) post-injury (from trauma to commencement of neuro-rehabilitation program) = 10.9 months (SD = 15.7); All subjects underwent rehabilitation program (average time = 10.53 months (SD = 6.24));	FIM+FAM Revised Scale (before and after treatment): FPA, FPD, and PFG	Driver status: Driver (patients who drove despite strong and repeated recommendations from the Center to desist from doing so when they began the rehabilitation program) and non-driver (patients not driving at the time they began the	Single cohort study design; Retrospective	35.3% of patients reported driving at time of admission (against doctor recommendations);70.6% of patients reported driving upon discharge; Significant differences found in all of the functional areas (self-care, sphincter control, mobility, locomotion, communication, psychosocial adjustment, cognitive functions, mean total scale, and gain at discharge.) of the FIM + FAM-Revised Scale between the driver and non-driver group at the	Class III: Retrospective; N < 100; Single cohort; Self-report Conclusion: Patients with physical functionality above 80% returned to driving and are at increased risk for driving incidents; however, neurorehabilitation can improve the rate of safe return to driving

		Recruited from Center for Brain Injury Rehabilitation		rehabilitation program although they had a pre-injury driver's license)		beginning of treatment (rehabilitation program, $p < 0.01$), but not after treatment; Physical functionality (self-care, communication, sphincter control, transfer, and locomotion) score above 80% (functional normality starts at around 80%) seems to be the best predictor of a driver insisting to return to drive, irrespective of cognitive and/or emotional deficits, and against doctor recommendations	
Return to driving within 5 years of moderate-severe traumatic brain injury Novack et al. (2010) [19] USA Funded by the National Institute on Disability and Rehabilitation Research	To examine variables associated with return to driving after TBI	1 year (N = 5942; age (mean) = 38.0 years (SD = 17.8); 73.2% male and 26.8% female), 2 years (N = 4,628), and 5 years (N = 2,324); Patients with TBI (predominantly moderate-severe patients who attended acute rehabilitation); Patients enrolled in the TBI Model System national database	GCS score; DRS; FIM; SWLS; Demographic variables	Driving status at each follow-up interval (1, 2, and 5 years): driving (if respondents indicated 'drives vehicle' as primary mode of transportation) and non-driving (if respondents indicated 'rides with someone else, public transit, special bus or van service and N/A-no motorized vehicle' as primary means of transportation)	Single cohort study design; Retrospective	42.0% of participants were driving at 1 year (45.5% with low injury severity and 36.4% with high), 48.9% of participants at 2 years (50.5% with low injury severity and 45.6% with high), and 53.0% of participants at 5 years (51.9% with low injury severity and 49.1% with high); Less severe TBI (quantified by FIM at discharge, DRS at discharge, or injury severity level) associated with odds of driving (FIM (10 point increase) OR=1.42, 95% CI = 1.38 to 1.45, DRS (5 point decrease) OR = 3.03, 95% CI = 2.76 to 3.32, and injury severity (low vs. high) OR = 1.26, 95% CI = 1.13 to 1.40); Driving associated with higher life satisfaction at each follow-up year ($p < 0.0001$); Functional status at	Class III: Retrospective; N > 100; Self-report Conclusion: Majority (53%) of participants with TBI returned to driving within 5 years after brain injury; however, less severe TBI predicted faster return to driving

						rehabilitation discharge and demographic variables (age at injury, race, pre-injury residence, pre-injury employment status, and education level) associated with odds of driving (1.52 (95% CI = 1.39 to 1.67) times greater at 2 years post-injury than at 1 year post-injury, 1.19 (95% CI =1.05 to 1.35) times greater at 5 years post-injury than at 2 years post-injury and 1.81 (95% CI = 1.59 to 2.07) times greater at 5 years post-injury than at 1 year post-injury	
Role of premorbid factors in predicting safe return to driving after severe TBI Pietrapiana et al. (2005) [20] Italy Funded by Ordine Mauriziano di Torinoto to Marco Tamietto	To examine factors that predict post-injury fitness to drive in patients with severe TBI	N = 66 pairs; Patients with severe TBI; 54 men and 12 women; Age (mean) of patients = 34.3 years (SD = 9.4); Time (mean) post-injury = 5.6 years (SD = 3.7); GCS (mean) score = 5.9 (range 3 to 8); Recruited from rehabilitation program; Caregivers	Patient: GCS score; LOC; FIM + FAM (administered at discharge from Rehabilitation Centre); VST (three trials of visual cancellation tasks; yielded an overall attention score); WAIS-R SDS (number of digits correctly replaced within 90 s); Caregiver: Pre-TBI-risky-personalit y index of patient (rated indolence, impulsiveness, calmness, irritability, sociability, aggressiveness, and tendency to inattention; yielded a global index score); Pre-TBI-risky-driving-st yle index of patient (rated caution, tendency to inattention,	Driving status: driving (n = 31, driving for at least a year after TBI) and non-driving (n = 35); Driving safety (complete driving record, number of post-TBI car accidents and violations, collected in interview with caregiver; composite score was created)	Cohort study design; Retrospective	31 (47%) of patients with TBI returned to driving; Groups did not significantly differ on demographic and biographic driving-related variables (e.g., age at license); Compared to non-driver group (N = 35), the driver group had shorter coma duration (LOC; P = 0.042); GCS scores between groups did not differ significantly; 35.5% (N = 11) of patients who resumed driving were subsequently involved in a car accident, while remaining 64.5% (N = 20) were not; Model (hierarchical multiple regression model) with years post-injury, pre-TBI accidents and violations (from driving record), pre-TBI-risky-personality-in	Class III: Retrospective; N < 100; Self-report Conclusion: LOC was the main parameter that distinguished between drivers and non-drivers; Predictors of driving safety included years post-injury, pre-injury accidents and violations, pre-TBI-risky-personali ty-index, and pre-TBI-risky-driving-s tyle-index

			competitiveness, observance of the road traffic riles, and reckless behaviour; yielded a global index score)			dex, and pre-TBI-risky-driving-style-index accounted for 72.5% of the variation in the outcome measure, driving safety (number of post-TBI car accidents and violations, $p < 0.01$)	
Barriers to driving and community integration after TBI<br>Rapport et al. (2006) [21]<br>USA<br>Funded by the National Institute on Disability and Rehabilitation Research	To examine driving status, perceived barriers to return to driving, and community integration outcomes (perceived quality of community integration and participation)	N = 51; 6 women and 45 men; Age (mean) = 39.1 years (SD = 13.3); Time (range) post-injury = 6 months to 10 years; GCS (mean) score at admission = 8.8 (SD = 4.4); 74.5% individuals with moderate to severe TBI; Recruited from participants of the Southeastern Michigan TBI Systems database	BDQ; CIM, Craig Hospital Assessment and Reporting Technique - Short Form; GCS score; PANAS; SPS	Driving status (drivers vs. non-drivers)	Single cohort study design; Cross-sectional	39% of individuals with TBI had returned to driving; GCS and age were unrelated to driving status; Social barriers significantly predicted driving status (OR = 3.30), followed by BDQ resources (OR = 1.66), physical (OR = 1.24), cognitive (OR = 1.07), and psychological (OR = 0.84) domains; Total logistic regression model was significant ($p = 0.025$); BDQ and SPS accounted for unive variance (sr^2 = 0.14 and 0.15, respectively); Age, education level, and GCS were unrelated to community integration outcomes; Significant difference in community integration measures (e.g., Craig Hospital Assessment and Reporting Technique Social Mobility), indicating greater integration among drivers, compared to non-drivers;	Class III: Cross-sectional; N < 100; Self-report<br>Conclusion: Individuals with TBI who return to driving have better community integration; however, social barriers accounted for most of the variance in driving status
On the road again after traumatic brain injury: driver safety and behaviour following on-road assessment and rehabilitation	To examine driver safety and behaviour following on-road assessment and	N = 106; 81 (76%) male; Patients with TBI; 2.05% (PTA, 39.1% if GCS) mild, 25.5% (PTA, 17.5% if GCS) moderate,	Medical records (including GCS score and PTA duration)	Self-reported driver behaviour and safety (including frequency of	Cohort study design; Retrospective; Two groups (pass group included those who returned to driving	In rehabilitation group, TBI was more severe (compared on PTA duration and GCS score) than pass group; No significant difference between pre/post-injury	Class III: Retrospective; N > 100; Self-report<br>Conclusion: Drivers with TBI, after driver assessment and rehabilitation, report

Ross et al. (2016) [22] Australia Funding not described	rehabilitation after TBI	and 36.3% (43.4% if GCS) severe; Recruited from previous study (rehabilitation program)		driving, distances driven, perceived importance of driving, driving conditions avoided, etc.)	after one on-road assessment (without driving lessons) and the rehabilitation group which required more than one on-road assessment and driver rehabilitation lessons)	crash rates between pass and rehabilitation group; When compared to pre-TBI, drivers reported limiting driving time (36.8%), drove more slowly (40.6%), greater difficulty with navigating (41.5%), and near crashes (20.0%); Rehabilitation group, compared to pass group, significantly more likely to drive less frequently, for shorter distances, and to avoid driving with passengers, busy traffic, night and freeway driving	changing driving behaviour, but do not report more crashes
Utility of the UFOV test with mild traumatic brain injury Schneider and Gouvier (2005) [23] USA Funding not described	To examine the use of the UFOV in individuals with mTBI	N = 80; Individuals with TBI (N = 40); Age (mean) of TBI group = 22.0 (SD = 4.1); mTBI (90% of participants using LOC and 77.5% using PTA); Time (mean) post injury = 7.1 years (SD = 5.1); Control group (n-40); Recruited from the undergraduate population	UFOV (subtest 1, 2, and 3, measuring central vision, divided attention, and selective attention; composite score, ranging from 1 (low risk) to 5 (high risk)); TMT A (time); TMT B (time); Processing Speed Index from WAIS-III; SDMT	Crash status in the past two years (self-report, number of accidents and traffic citations)	Quasi-experimental case-control design; Retrospective	No significant difference on any measure between very mild, mild, and moderate to severe TBI); Significant difference in the number of reported accidents between TBI group and control, where number of accidents was greater for those with TBI ($p < 0.05$), but not significant for number of citations; Scores on UFOV 3 were not significantly correlated with crash group for both groups (TBI and control group) or with very mild, mild, or moderate to severe TBI participants; TMT A and B were not predictive of self-reported crashes or citations in individuals with mild TBI	Class III: Quasi-experimental case-control design; Retrospective; N < 100; Driving simulator Conclusion: Neither UFOV or other neuro-psychological measures correlated with crash status
Driving behaviors following brain injury: Self-report and motor vehicle records	To examine driving behaviours after TBI using	N = 69; 47 individuals with TBI: Time (mean) post injury = 4.6	TBI	Driving status, driving record (reported and unreported	Cohort study; Retrospective design; With control group	Results did not show a statistically significant difference in aberrant driving behaviours between	Class III: Retrospective; N < 100; Subjective and objective measures Conclusion: No

Schultheis et al. (2002) [24] USA Funded by the Charles Edison Foundation and by the National Institute of Child Health and Human Development	both subjective and objective outcome measures	years (SD = 4.6); Recruited from individuals who had successfully completed a driver re-evaluation; 22 healthy controls (matched for age, gender, education, and years of driving experience)		accidents) and unsafe driving situations (such as speeding, driving while intoxicated, driving while overtired, etc.)		group with TBI and group without	evidence to support persons with TBI change driving behaviours five years post TBI, suggesting TBI drivers are able to safely integrate into the driving community

Note. ACT = Auditory Consonant Trigrams; BDQ = Barriers to Driving Questionnaire; CIM = Community Integration Measure; CPT-II = Continuous Performance Test II; CTT = Colored Trails Tests; DAS = Driver assessment scale; DASS-21 = Depression Anxiety Stress Scales; DBQ = Driver Behaviour Questionnaire; D-KEFS = Delis-Kaplan Executive Function System; DPI = Driving Performance Inventory; DRS = Disability Rating Scale; FAM = Functional assessment measure; FIM = Functional Independence Measure; FPA = Functionality percentage at admission; FPD = Functionality percentage at discharge; GCS = Glasgow Coma Scale; GOAT = Galveston Orientation and Amnesia Test; GRS = Global rating scale; HPT-HR = Hazard perception hit rate; HPT-RT = Hazard perception response time; HPT = Hazard Perception Test; LOC = Length of coma; LogMar = Logarithm of minimum angle of resolution; MMSE = Mini Mental State Examination; mTBI = Mild traumatic brain injury; MVPT = Motor free visual perception test; OT-DHMT = Occupational therapy-driver off road assessment; PANAS = Positive and Negative Affectivity Scale; PCRS = Patient competency rating scale; PFG = Percentage of total functional gain; PTA = Posttraumatic amnesia; RAVLT = Rey Auditory Learning Test; RCFT = Rey Complex Figure Test; RLRCT = Road law road craft test; RT = Reaction time; SD= Standard Deviation; SPI = Simulator Performance Index; SPS = Social Provision Scale; SSRT = Simple spatial reaction time; SWLS = Satisfaction with Life Scale; TBI = Traumatic brain injury; TBIMS: Traumatic Brain Injury Systems; TMT = Trail Making Test; TMT A = Trail Making Test A; TMT B = Trail Making Test B; UFOV = Useful field of view; UQ-HPT = University of Queensland-hazard perception test; WAIS = Wechsler Adult Intelligence Scale.

References

1. Korteling, J.E.; Kaptein, N.A. Neuropsychological driving fitness tests for brain-damaged subjects. *Arch. Phys. Med. Rehabil.* **1996**, *77*, 138–146.
2. Lew, H.L.; Poole, J.H.; Jaffe, D.L.; Huang, H.C.; Brodd, E. Predictive validity of driving-simulator assessments following traumatic brain injury: A preliminary study. *Brain Inj.* **2005**, *19*, 177–188.
3. McKay, A.; Liew, C.; Schonberger, M.; Ross, P.; Ponsford, J. Predictors of the on-road driving assessment after traumatic brain injury: Comparing cognitive tests, injury factors, and demographics. *J. Head Trauma Rehabil.* **2015**, in press.
4. Neyens, D.M.; Boyle, L.N.; Schultheis, M.T. The effects of driver distraction for individuals with traumatic brain injuries. *Hum. Factors* **2015**, *57*, 1472–1488.
5. Novack, T.A.; Banos, J.H.; Alderson, A.L.; Schneider, J.J.; Weed, D.; Blankenship, J.; Salisbury, D. UFOV performance and driving ability following traumatic brain injury. *Brain Inj.* **2006**, *20*, 455–461.
6. Ross, P.E.; Ponsford, J.L.; di Stefano, M.; Charlton, J.; Spitz, G. Predictors of on-road driver performance following traumatic brain injury. *Arch. Phys. Med. Rehabil.* **2015**, *96*, 440–446.
7. Stokx, L.C.; Gaillard, A.W.K. Task and driving performance of patients with a severe concussion of the brain. *J. Clin. Exp. Neuropsychol.* **1986**, *8*, 421–436.

8. Beaulieu-Bonneau, S.; Fortier-Brochu, E.; Ivers, H.; Morin, C.M. Attention following traumatic brain injury: Neuropsychological and driver simulator data, and association with sleep, sleepiness, and fatigue. *Neuropsychol. Rehabil.* **2015**, *24*, 1–23.
9. Gamache, P.L.; Lavailliere, M.; Tremblay, M.; Simoneau, M.; Teasdale, N. In-simulator training of driving abilities in a person with a traumatic brain injury. *Brain Inj.* **2011**, *25*, 416–425.
10. Preece, M.H.W.; Horswill, M.S.; Geffen, G.M. Driving after concussion: The acute effect of mild traumatic brain injury on drivers hazard perception. *Neuropsychology* **2010**, *24*, 493–503.
11. Preece, M.H.W.; Horswill, M.S.; Geffen, G.M. Assessment of drivers' ability to anticipate traffic hazards after traumatic brain injury. *J. Neurol. Neurosurg. Psychiatr*. **2011**, *82*, 447–451.
12. Baker, A.; Unsworth, C.A.; Lannin, N.A. Fitness to drive after mild traumatic brain injury: Mapping the time trajectory of recovery in the acute stages post injury. *Accid. Anal. Prev.* **2015**, *79*, 50–55.
13. Coleman, R.D.; Rapport, L.J.; Ergh, T.C.; Hanks, R.A.; Ricker, J.H.; Millis, S.R. Predictors of driving outcome after traumatic brain injury. *Arch. Phys. Med. Rehabil.* **2002**, *83*, 1415–1422.
14. Cullen, N.; Krakowski, A.; Taggart, C. Early neuropsychological tests as correlates of return to driving after traumatic brain injury. *Brain Inj.* **2014**, *28*, 38–43.
15. Hawley, C. Return to driving after head injury. *J. Neurol. Neurosurg. Psychiatr.* **2001**, *70*, 761–766.
16. Ilie, G.; Mann, R.E.; Ialomiteanu, A.; Adlaf E.M.; Hamilton, H.; Wickens, C.M.; Ashbridge, M.; Rehm, J.; Cusimano, M.D. Traumatic brain injury, driver aggression and motor vehicle collisions in Canadian adults. *Accid. Anal. Prev.* **2015**, *81*, 1–7.
17. Labbe, D.R.; Vance, D.E.; Wadley, V.; Novack, T.A. Predictors of driving avoidance and exposure following traumatic brain injury. *J. Head Trauma Rehabil.* **2014**, *29*, 185–192.
18. Leon-Carrion, J.; Dominguez-Morales, M.R.; Martin, J.M. Driving with cognitive deficits: Neurorehabilitation and legal measures are needed for driving again after severe traumatic brain injury. *Brain Inj.* **2005**, *19*, 213–219.
19. Novack, T.A.; Labbe, D.; Grote, M.; Carlson, N.; Sherer, M.; Arango-Lasprilla, J.C.; Bushnik, T.; Cifu, D.; Powell, J.M.; Ripley, D.; et al. Return to driving within five years of moderate-severe traumatic brain injury. *Brain Inj.* **2010**, *24*, 464–471.
20. Pietrapiana, P.; Tamietto, M.; Torrini, G.; Mezzanato, T.; Rago, R.; Perino, C. Role of premorbid factors in predicting safe return to driving after severe TBI. *Brain Inj.* **2005**, *19*, 197–211.
21. Rapport, L.J.; Hanks, R.A.; Bryer, R.C. Barriers to driving and community integration after traumatic brain injury. *J. Head Trauma Rehabil.* **2006**, *21*, 34–44.
22. Ross, P.; Ponsford, J.L.; Stefano, M.D.; Charlton, J.; Spitz, G. On the road again after traumatic brain injury: Driver safety and behaviour following on-road assessment and rehabilitation. *Disabil. Rehabil.* **2016**, *38*, 994–1005.
23. Schneider, J.J.; Gouvier, W.D. Utility of the UFOV test with mild traumatic brain injury. *Appl. Neuropsychol.* **2005**, *12*, 138–142.
24. Schultheis, M.T.; Mathis, R.J.; Nead, R.; DeLuca, J. Driving behaviors following brain injury: Self-report and motor vehicle records. *J. Head Trauma Rehabil.* **2002**, *17*, 38–47.
